# The Earlier, the Better? An In-Depth Interview Study on the Ethics of Early Detection with Parents of Children at an Elevated Likelihood for Autism

**DOI:** 10.1007/s10803-023-06139-8

**Published:** 2023-09-26

**Authors:** Gert-Jan Vanaken, Ilse Noens, Jean Steyaert, Lotte van Esch, Petra Warreyn, Kristien Hens

**Affiliations:** 1 Centre for Ethics, Department of Philosophy, University of Antwerp, Antwerp, Belgium; 2 Parenting and Special Education Research Unit, Faculty of Psychology and Educational Sciences, KU Leuven, Leopold Vanderkelenstraat 32, Leuven, 3000 Belgium; 3 Leuven Autism Research (LAuRes), KU Leuven, Leuven, Belgium; 4 Centre for Developmental Psychiatry, Department of Neurosciences, KU Leuven, Leuven, Belgium; 5 Research in Developmental Disorders Lab, Department of Experimental Clinical and Health Psychology, Ghent University, Ghent, Belgium; 6 Institute of Philosophy, KU Leuven, Leuven, Belgium

**Keywords:** Early detection, Autism, Neurodiversity, Ethics, Parents

## Abstract

Autism is increasingly viewed as an expression of neurodiversity deserving accommodation, rather than merely as a disorder in need of remediation or even prevention. This reconceptualization has inspired calls to broaden the ethical debate on early autism care beyond matters of efficient screenings and effective interventions. We conducted 14 in-depth interviews with 26 parents of infants at an increased likelihood for autism (siblings, preterms and children with persistent feeding difficulties) to understand which benefits and risks these parents see for the implementation of a systematic, early autism detection program in our region. With this study, we aim to contribute empirically to the ethical debate on good and just early autism care in the age of neurodiversity. Data were analyzed according to the QUAGOL-methodology. Three main themes emerged from our analysis. In their evaluation of early autism detection, parents discussed how a diagnosis helps gain a different perspective fostering understanding and recognition for both child and parent. Second, a diagnosis supports parents in adjusting their parenting practices, to justify this deviation from “normal” parenting and to strive for such adjusted environments beyond the nuclear family. Third, an autism diagnosis induces ambiguities parents need to navigate, involving questions on whether and when to mobilize the diagnostic label and which language to use to talk about autism. We discuss the complex position of parents of a (potentially) autistic child in terms of moving back and forth across the ab/normal binary and describe implications for the ethical debate on early autism detection.

**Supplementary Information:**

The online version contains supplementary material available at 10.1007/s10803-023-06139-8.

## Introduction

In past decades, parents of a potentially autistic^1^ child have been encouraged to engage in the earliest possible diagnostic assessment and intervention for autism (Zwaigenbaum et al., 2015). Yet, nowadays autism is increasingly viewed as an expression of neurodiversity deserving accommodation, rather than merely as a disorder in need of remediation or even prevention (Pellicano & den Houting, 2022). This changing conceptualization brings new questions to the fore on what is best to do for parents of (potentially) autistic children in these early life stages. Also, clinical practitioners, researchers and public health services might need to reconsider the goals and methods of early autism support (Brown et al., 2021; Leadbitter et al., 2021; Mottron, 2017; Schuck et al., 2022). Put differently, it is a pressing and valuable task for the autism field to rethink what *good and just* early autism care looks like in the age of neurodiversity. In what follows, we will briefly introduce the current academic debate on early autism detection, specify which new questions have come to the surface recently in this regard, and argue how this in-depth interview study with parents of potentially autistic children can help answering these questions.

Up to now, there has been a fairly broad consensus among autism scientists that early detection, diagnosis and intervention for autism are the way forward in optimizing care for autistic children and their relatives (French & Kennedy, 2018; Green & Garg, 2018; Magán-Maganto et al., 2017). Early detection and diagnosis of autism indeed provide an entry ticket to various services, such as early psychosocial intervention programs. Compared to interventions in childhood and adolescence, programs offered in the first years of life are expected to be more effective in supporting the child’s development. The rationale here is that such early interventions would “capitalize on experience-dependent neuroplasticity” and would enrich the “diminished, unelaborated, and truncated social and communication learning opportunities” (sic.) of autistic infants (Landa, 2018, pp. 25–26). Therefore, the chief questions for the field have revolved around matters of accuracy and effectiveness of these early autism programs (Hickey et al., 2021). For example, which detection instruments predict an autism diagnosis most accurately at an early age? How can an overly amount of false positive and, perhaps specifically, false negative screenings be avoided (Guthrie et al., 2019)? Which early interventions provide robust and large enough effect sizes to justify the effort and cost of their implementation as public health program (Sandbank et al., 2020)?

When looking at other public health ethics discussions, we see, however, that deciding on the rights and wrongs of early detection and intervention programs has often involved more than weighing operational risks and benefits. For example, discussions on prenatal screening for Down syndrome and screenings and early treatments for breast cancer have spotlighted fundamental questions on drawing lines between health and disease, on living well beyond the boundaries of a “normal” body and mind, and on reproducing structural discrimination of disabled people despite practitioners’ good intentions (Parens & Asch, 2003; Rogers, 2019).

Autistic scholars and neurodiversity proponents have raised similar conceptual questions in autism research over the past years. These questions include whether we can conceive of autism beyond a clinical diagnosis or neurodevelopmental disorder in need of treatment, and what legitimate goals and targets are for clinical support (Ne’eman & Pellicano, 2022). For autistic adults, interventions are already increasingly modelled on neurodiversity claims of acceptance of difference and accommodation of the environment, such as generating adapted workplaces and sensitizing colleagues about autism (Lai et al., 2020). With some notable exceptions (Fletcher-Watson, 2018; Leadbitter et al., 2021; Schuck et al., 2022), applications of the neurodiversity paradigm are, however, still largely unexplored terrain when it comes to young children and the sphere of early detection and intervention (Savarese, 2010).

Recently, some scholars have called for a broader reflection on such conceptual issues when developing early autism detection and intervention programs (Lai et al., 2020 (Annex 1); Manzini et al., 2021). In their agenda-setting review “The Ethics of Autism”, Hens et al. (2019) identified key clusters of autism-related ethical questions. One of those clusters concerns questions on parental rights and duties about obtaining an early autism diagnosis and pursuing interventions for their child. For example, can parents decline a diagnostic assessment? Is it the parents’ duty to aim for optimal (or “normal”?) functioning of their child through interventions? Or should parents instead accept and accommodate their child’s autism as a neutral, neurological difference?

Apart from such calls to broaden the ethical debate, much of the actual work still needs to be done to reshape clinical practices oriented towards young autistic children and their relatives. Recently, some valuable theoretical contributions to this ethical debate have emerged (Brown et al., 2021; Chapman & Botha, 2022; Leadbitter et al., 2021; MacDuffie et al., 2021; Schuck et al., 2022). One of the authors of this manuscript (GJV) contributed as well to this debate by analyzing early autism interventions with a disability-sensitive interpretation of the concept of vulnerability. Vanaken (2022b) theorized that early autism interventions do not need to be set aside as mere reproductions of the pathology paradigm of autism. Yet, he argued that these care practices could be remodeled around obligations of solidarity and empowerment and therefore be reclaimed as spaces for political contestation contributing to the social change that neurodiversity proponents call for.

Empirical work on the ethics of early autism care is, however, still scarce. Therefore, we are convinced it is essential to explore the viewpoints and experiences of autistic people and their relatives regarding these topics. They are indeed directly involved actors bringing valuable knowledge and lived experiences to the discussion table (Newell, 2006). The interview study we present here explicitly aims to contribute empirically to the debate by understanding how parents of (potentially) autistic infants think about early autism care^2^. Our research questions were twofold: (1) Which advantages and risks do parents of a child at increased likelihood for autism see for clinical implementation of an early detection program in Flanders, Belgium? (2) How do they experience their role as a parent of a young infant being tracked for autism characteristics?

## Methods

The description of our methodology is based on the 32-item, “Consolidated criteria for reporting qualitative studies” (COREQ) (Tong et al., 2007) and the “Key criteria for successful submissions of qualitative manuscripts to JADD” (van Schalkwyk & Dewinter, 2020).

### Participants

We conducted 14 semi-structured, in-depth duo interviews with 26 parents of 14 children taking part in the Tracking Infants At Risk for Autism study^3^. TIARA is a prospective, longitudinal cohort study on the development of children at an increased likelihood for autism between the age of 5 and 36 months. The study includes siblings of children with an established autism diagnosis, infants born prematurely under 30 weeks of gestation and infants with persistent, medically insufficiently explained feeding problems^4^.

In our interview study, these three groups are represented as follows by the 14 parent couples: prematurely born children (*n* = 3), children with medically unexplained feeding problems (*n* = 4) and children with an older autistic sibling (*n* = 7). The gender, educational attainment and reported ethnicity of interviewed parents are presented in Table 1. Children were between 11 and 16 months old when the interviews occurred (corrected age for preterms). Parents were asked to participate in the interview study during their visit to the TIARA baby lab^5^. In case of interest, their contact details were passed to the first author. Twenty parent couples were contacted in total of which two did not respond and four declined mainly due to time constraints.

Our interest in these parents’ opinions and experiences stems from their unique position as TIARA-participants. First, these parents’ children have been labelled “at-risk” for autism without being necessarily concerned themselves about their child being on the autism spectrum, which is an exceptional experience. Second, as they chose to take part in an early detection study, these parents might resemble well future early adopters of early detection programs in our region.

The interviewees differed in terms of their experiences with autism. Answers and stories shared by parents of the sibling group were primarily based on their lived experiences in parenting an older, autistic child. Parents from the feeding difficulties and preterm groups, however, were not entirely naive in their responses either. Many of them also had some relevant experiences with autism, be it in professional settings, extended family contexts or in their circle of friends. Two parent couples explicitly stated not to have any experiences with autism beyond some general ideas circulating in the public sphere (Interviews (IV) 3, 12). None of the interviewees self-identified as autistic. For these reasons, we indicated parents’ relevant autism experiences as well in Table 1 and, when relevant, these experiences are spelled out too in the Results section, indicating when parents shared a personal experience versus an expectation which did not directly rely on first-hand experience with autism.

Throughout the interviews, none of the parents expressed worries about their child at the time of interviewing, despite being aware of the increased theoretical likelihood for their child to be on the autism spectrum. For two parent couples in the feeding difficulties (IV 11) and preterm group (IV 12), the initial recruitment into the early detection protocol took them by surprise and caused some stress. These two couples reported during their respective interviews that the experience of the child’s development being followed up during the study visits, having reassuring conversations with the study’s researchers seeing their child developing as expected, made them feel more comfortable over time. Other parents in the preterm group referred to increased likelihoods for a variety of medical and developmental conditions as a factor which did not make them worry particularly about autism. In the sibling group though, parents did elaborate on their heightened awareness about potential autistic features in their youngest child, as they often compared to the sibling with autism.

### Data Collection and Analysis

All fourteen interviews were conducted in Dutch by the first author in a face-to-face home setting with both parents, where possible (see Table 1). Two students (Master of Medicine) participated in the first five interviews as part of their master’s thesis. We obtained written informed consent from each participating parent (Ethics Committee Research KU Leuven, S61507) and provided a brief oral introduction stating that we were interested in parents’ opinions on the benefits and risks of clinically implementing early autism detection in Flanders, Belgium. JS and KH developed a semi-structured interview topic guide (see Annex 1) based on their respective experiences as senior clinical practitioner and autism researcher and as philosopher and (bio)ethicist. This guide remained unchanged after a mock interview. The duration of the interviews ranged between 50 and 105 min with an average of 75 min per interview. Interviews with parents from the sibling group tended to last longer than those with parents from the preterm and feeding difficulties groups, probably because they had more autism-related experiences to draw from. Interviews were audio recorded and fully transcribed verbatim using *f4* software. All names (people, schools, etc.) were pseudonymized in the transcripts. Transcripts were not sent back to participants. Data collection and first steps of data analysis were done in parallel in order to define when new interviews did not add up anymore to the existing data, this is when we noted that no significantly new themes were discussed in additional interviews.

We employed the Qualitative Analysis Guide of Leuven (QUAGOL) to analyze our data (Dierckx de Casterlé et al., 2012). The QUAGOL guide is a comprehensive and systematic approach to qualitative data analysis mainly embedded within Grounded Theory and consisting of two parts: a preparatory, inductive phase leading up to a list of codes and a more deductive phase including actual coding and analysis of the emerging concepts. In this first part of the analysis following QUAGOL, the first author made a narrative, one-page summary of each interview, staying close to the participants’ words and phrasings. Next, each *narrative report* was developed into a *conceptual report* by rephrasing and restructuring them in a more abstract and schematic way. The other team members listened to or read the original interviews to verify whether these conceptual reports captured the essential elements of each interview in relation to the research questions. Adjustments to these reports were made during regular team meetings. A cross-case analysis of the 14 conceptual reports led to a list of 20 codes which we briefly described in two or three sentences based on our understanding at that point. In the second part of the analysis, we used NVivo12 to code the transcripts with our inductively derived code list while making memos throughout this process. Based on the fragments assigned to each code, we fleshed out our understanding of codes, becoming “concepts” described each in 200–500 words. Lastly, we integrated these well-described concepts in an overarching storyline, checking back with the conceptual reports to verify this reflected the most relevant parts of the data, including both majority and minority views and opinions.

### Researchers’ Background and Theoretical Stance

In accordance with guidelines on reporting on qualitative studies in the field of autism research, we also want to provide some background information on us as researchers. At the time of the study Gert-Jan Vanaken is a medical doctor and PhD candidate working on the ethics of early autism care. He combines empirical, qualitative work with theoretical reflections at the crossroads of disability studies and bioethics. He has a particular interest in contributing to the development of neurodiversity-affirmative autism care practices. Ilse Noens is professor in educational sciences and chair of the *Leuven Autism Research consortium*. She conducts participatory research on parenting and effective psychosocial support for autistic people. Jean Steyaert is professor in child & adolescent psychiatry and head of clinic at the Expertise Centre for Autism at the University Hospitals Leuven. His research focuses on early autism detection and biomedical autism interventions. Petra Warreyn is assistant professor in clinical psychology. Her work mainly focusses on the early development of and care for children with or at elevated likelihood for autism or learning disabilities, taking into account contextual factors. Lotte van Eschis a postdoctoral researcher involved in coordinating the TIARA-study and she has previously conducted both quantitative and qualitative research on parenting autistic children. *[name blinded for review]* is a research professor in bioethics and co-founder of the Autism Ethics Network. She focuses among other things on ethical and conceptual questions about developmental diversity and psychiatric diagnoses.

Theoretically, we commit to a neurodiversity perspective on autism research (Bertilsdotter Rosqvist et al., 2020), embedded within a critical realist philosophy of science (Botha, 2021; Kourti, 2021). Critical realists deviate from positivists, interpretivists and constructivists as they combine a *realist* ontological stance with *relativist* epistemological positions (Bhaskar & Danermark, 2006). Concretely, we assume that autism, as a phenomenon of shared lived experiences (Hens, 2021), truly exists and is not merely a social construction. Yet, precisely because it is linked to individual, albeit shared, experiences, knowledge about autism is always approximative and fallible. On this account, exploring first-hand experiences of autistic people and their close relatives may be a tangible way to reach more ‘objective’ knowledge about autism. Though, for critical realists, such objectivity is not synonymous to ‘neutrality’. Knowledge can be at the same time more objective and less (politically and ethically) neutral. Put differently, empirical findings and observable facts entail ethical dimensions (Kourti, 2021). For example, phenomenological research on stimming among autistics has highlighted such behaviors serve a multitude of potentially valuable purposes, ranging from emotional coping to experiencing pleasure (Kapp et al., 2019). This type of research has improved ‘objective’ understanding of autistic behavior, while having normative implications: stimming cannot be easily seen any more as a legitimate target for normalizing clinical interventions without considering its value for the person in case. Critical realism’s combination of ontological realism, epistemic relativism and its specific take on the relation between facts and values justify why ethical reflection (on matters such as early autism care) necessarily requires empirical research engaging with lived experiences and opinions of most affected groups (Archer et al., 2016). Such empirically based ethical research is what we aim to contribute to with this study.

When it comes to our take on a neurodiversity perspective to autism research, we make the following assumptions. We conceptualize autism firstly as a morally neutral expression of the wide, cognitive, emotional, behavioral diversity in how people experience and engage with the world (Walker, 2014). We commit to the stance that problems experienced by autistic people cannot be merely thought of as deficits situated within individual bodies and minds. Rather, they emerge from an interaction of neurocognitive differences with social and societal contexts that are often ill-accommodated to the needs of autistic people (Dwyer, 2022). We situate autism primarily on the experiential level and we focus on the interactions between individuals and their environment to understand experienced difficulties. Therefore, first-hand, lived experiences of autistic people and their close relatives are primary sources to produce relevant, ‘objective’ autism knowledge (Chapman, 2022; Gillespie-Lynch et al., 2017). Furthermore, taking a neurodiversity perspective on autism research also implies that we consider our role as researchers as academic allies to the neurodiversity movement, striving to contribute to social justice for and emancipation of autistic people (Vanaken, 2022a). Concretely, our neurodiversity perspective will become particularly tangible in the discussion session of this paper.

## Results

We structured the concepts arising from our analysis into an overarching storyline entailing three themes which are presented below: (1) gaining a different perspective after a diagnosis, (2) parenting differently, and (3) navigating the ambiguous aspects of an autism diagnosis.

### Theme 1: Gaining a Different Perspective

When reflecting about the potential *value* of an autism diagnosis, nearly all parents extensively talked about aspects of improved understanding and recognition as two direct, beneficial consequences of such a diagnosis.

Parents across three groups expected or experienced that the knowledge and information that comes with an autism diagnosis (would) help them understand better how their child feels, thinks and reacts. Some parents described it as putting up a different pair of glasses to look at the child and be more empathizing and comprehending regarding behaviors they would have otherwise not understood.

A parent couple from the sibling group (IV 2) said the following about their oldest autistic son:

Mother: *“If we had already looked at him from that perspective as a baby, it would have spared him quite some trouble. If we had noticed back then that there was a link between him being irritable and going to that busy fair the day before, well… But that is not how you view things then. You only see your child is unwell and you wonder why. If we could have viewed him through a different pair of glasses back then…”.* Father: *“…then we would have understood him a whole lot better.”*

Such improved understanding is not only expected to be helpful for the child, but also for parents themselves in order to feel less frustrated, powerless or uncertain about their parenting skills. A mother (IV 1) who has an autistic son with a co-occurring intellectual disability shares the following about a potential diagnosis for her younger daughter:

Of course, it will still be a quest (…), but at least you won’t be frustrated, or so frustrated, because you have a frame of reference. Whereas, if you don’t know anything, my experience is that you are simply hitting the wall.

Next to improved understanding of their child’s functioning, parents expected or experienced that a formal autism diagnosis could also provide a sense of recognition, both for the child and for themselves. Parents reported or feared that when their child presented atypical development features or behavior, third parties such as friends, family and other caregivers did or would fail to appreciate this in a pre-diagnostic phase. Atypicalities were sometimes brushed away to reassure parents, but more often parents discussed how third parties attributed blame to the child itself or to parents. Blaming the child for its atypicalities manifests itself mostly via pejoratives, such as “naughty”, “annoying”, “feisty” or “spoiled”. Parents perceived or anticipated blame towards themselves in terms of having insufficient pedagogical skills and in unsolicited or inappropriate parenting advice.

According to parents, a formal diagnosis could or did provide recognition to the fact that their child indeed functions and develops differently compared to most children, without immediately attributing blame. Across the three groups of interviewees, parents indicated such recognition would or did help them to counter pejoratives or (implicit) accusations of poor parenting expressed by other family members, friends or caretakers.

A mother from the feeding difficulties group (IV 11) who had a late-diagnosed autistic sister herself, shared the following:

I do think it eases things if you can explain why your child is so upset, or why they act out in certain ways. So, people understand oh, that’s why, it’s not just an ‘annoying’ child. Because that was the stamp given to my sister. You know… I do think it is easier for people to understand when there is a ‘label’ -to say it that way, even though it’s maybe not the right word to use.

A father (IV 2) of four children, of whom two have an autism diagnosis said that *“as a parent of a child with autism, you sometimes seem to be the parent that didn’t educate his child. While at home, you are endlessly spending time moving things in a good direction. When you get the autism label, a sense of recognition comes along. Okay, you are doing your best and it is autism that is in play, and it is not, or not entirely, about the quality of your parenting. For me that is important.”*

### Theme 2: Parenting Differently

Father: *“At this very young age, between one and two years old, I think the main thing is to educate parents on how to engage with their child (…) rather than focusing on the child itself.”* (IV 10, preterms group).

Beyond the more cognitive aspects of improved understanding and recognition, the vast majority of parents across groups discussed the relation between obtaining an autism diagnosis for their young child and being facilitated to *do* things differently as a parent. This change involves attuning their parenting behaviors towards their child and striving for other caregivers to adjust their interactions to generate autism-friendly environments for their child to develop and grow up in.

### Parenting Adjustments, Competence and Deculpabilisation

In all fourteen interviews, parents shared how they expected or experienced that an autism diagnosis would help them reshape their pedagogical practices to accommodate their child’s needs. Among other examples, this included practices such as introducing more predictability and structure in their daily lives, using strategies to prevent and deal with meltdowns, generating less sensorially overwhelming environments and communicating in a more concrete and visually supported way. A father from the feeding difficulties group (IV 6) without much personal autism experience hypothesized the following:

Within the autism field, there are probably methodologies to improve parenting, instead of always being angry, having to ignore it, or not being aware of what is happening. (…) I can imagine when your child receives too many stimuli, when she is always overwhelmed in the supermarket, you can either be angry or not taking her there anymore, but maybe- and I am just thinking out loud, you can bring a thick pair of sunglasses. Those might be silly things that can avoid turning a futility into a drama, if you are aware of this at least.

Post-diagnostically, parents evidently kept experiencing day-to-day parenting challenges. However, parents from the sibling group described some beneficial, emotional aspects after adjusting their parenting behaviors. Compared to the pre-diagnostic phase characterized by uncertainty over their child’s development and their own parenting skills, parents mainly reported decreasing feelings of guilt and increasing perceptions of parenting competency.

Mother: *“We became very uncertain about ourselves, but thanks to this diagnosis and the support, he found himself again, he found rest, and we did so too at a record pace. It explained so much, in the sense that things were not our fault.” (…)* Father: *“It was just like, wow, we are doing just fine as parents, but our son is just different.”* (IV 7, siblings group).

Parents in the preterms and feeding difficulties groups did not elaborate much on this last topic, except for one mother from the preterm group, who did not have any particular experience with autism (IV 12).

I would feel guilty, if he could have benefited from additional support, such as early interventions. If you know about this in time, you can opt for it. But if you only know at a later point that he could have learned things earlier, then it would feel like we could have helped him better [than we did] actually.

### Justifying Difference

Although these relatively straightforward adaptations in parenting behaviors do not strictly require a formal autism diagnosis, parents in the sibling group often emphasized that obtaining the diagnosis served as a turning point after which they felt more *legitimized* to try out such new things while stopping certain practices that did not work well for them.

A father from the sibling group (IV 8) for whom the diagnosis of his oldest son made a big impact on his parenting behavior reflected on this legitimation as follows:

And once you get the diagnosis, it clicks, and things suddenly fall in place. It makes you deal much better with this story, which is definitely a psychological thing. Nothing actually changes, but because someone else defines what is going on, I was like: okay, yes! And then, it became much easier to determine how I reacted to him in our interactions.

Parents described how the diagnosis would or did support them to differentiate their approach between autistic and non-autistic children, to resist the idea they would need to be “*the tough parent that does not give in to their child*” (IV 13, preterms group) and to deviate from typical and oft-advised parenting strategies. Two parent couples from the sibling group said the following about this:

Mother: *Without any prior knowledge about autism, who would think: ah, you need to put some silly illustrations or pictures in the right order… No one thinks about that! You think about those stupid episodes of the Supernanny: ‘if you do this, you get that, and you may put a little sticker on your card’. But that does not help at all (laughs)!”* (IV 2).

Father *(siblings group): “We relied much on standard educational practices: putting him in the corner, giving time-outs. We already followed a Triple-P training, about positive parenting, which did not work at all for him.” (…)* Mother: *“Yes, you stick to the parenting patterns that you’ve been raised with yourself, and with that Triple P training and so.” (…)* Father: *“For years, we’ve been putting him in a corner [as a disciplinary measure], until that corner looked all brown from his dirty hands… until they [autism practitioners] told us that was pedagogically useless for children with autism. So, if you can detect autism earlier, that would be a lot easier.”* (IV 14).

Fathers from the preterms and feeding problems group with a limited experience with autism sometimes specified the need for justification in more detail. For them, making “*all kind of exceptions*” in their parenting practices could only by justified when the autism diagnosis was formalized. In case there would only be an increased likelihood or a suspicion of autism, efforts to adjust parenting practices could be superfluous and thus more difficult to justify.

As said, parents in the sibling group mentioned that an autism diagnosis for their oldest child functioned as a justification for adjusting their parenting practices. However, when it comes to a possible diagnosis for their youngest child, there were two strands of opinions within the sibling group. Some of these parents stated that they would want to let their child have a diagnostic assessment in any case, expressing the need for confirmation whether their child is autistic to parent adequately. For some other parents within the sibling group though, this need for an early diagnosis seemed to have dissipated to some extent as they already gathered experience in parenting an autistic child and diversified their view of “normal” parenting.

A mother from the sibling group (IV 14) reflected on the potential need for a diagnosis for her youngest child:

If our daughter would have been the first-born, we would already have done the tests probably. But now our boundaries have shifted because of our older son [with autism]. (…) Also, some adaptations we made for him, simply became common practice in our family, so maybe it [autism-related difficulties] will be less noticeable for her.

### Striving for Adjusted Environments Beyond the Nuclear Family

In addition to changes in parent-child interactions, many parents discussed the value of an autism diagnosis as a tool to strive for adjusted environments for their child beyond the nuclear family. The diagnostic label could provide parents with language and legitimation to communicate with family, friends, daycare workers, kindergarten teachers etc., to take steps towards an autism-friendly environment for their child.

*Father (IV 12, preterms group): Also, towards family… prejudices do exist, you know. People easily point the finger at others, saying something is wrong. But then [i.e., with a diagnosis], you can actually name what it is, so people can also learn to engage with him correctly, for example in child day care.”*

Most parents, in particular those from the sibling group with a practical experience in these matters, emphasized however that such efforts are often not self-evident. A lack of sufficient and relevant knowledge or stereotypical views about autism often stand in the way. This generates tensions and doubts among parents on whether and when it is favorable to disclose their child’s diagnosis. These kinds of ambiguities that are raised by an autism diagnosis are discussed in the next section.

### Theme 3: Navigating the Ambiguities of an Autism Diagnosis

Parents discussed how a formal autism diagnosis might help to foster increased understanding, can provide recognition for experienced difficulties and efforts, and can be a tool to strive for a more autism-friendly environment for the child. Various parents did, however, also discuss some more ambiguous aspects of obtaining an early autism diagnosis. Here, there are two subthemes: (1) mobilizing the diagnostic label: blessing or curse? and (2) doubts about “correct” terminology to speak about autism.

### Mobilizing the Diagnostic Label: Blessing or Curse?

In order to benefit from the understanding and recognition a diagnosis might provide, this diagnosis generally needs to be disclosed. Parents discussed several points of doubt on whether to mobilize the diagnostic label in certain situations. First, they described or anticipated that appropriate knowledge about autism in child daycare and at schools is often lacking. By consequence, even after obtaining an autism diagnosis and sharing this with other caretakers, parents reported or expected that this would not necessarily result in the expected accommodations. Many parents did express their hope that teachers in regular educational settings would learn more in their training about autism and other developmental conditions.

In addition, one father from the preterms group (IV 13), who has a limited personal experience with autism and who works as a teacher himself, shared his worries about teachers adapting their practices merely based on the child’s diagnostic label:

*“This (sharing of the diagnosis) is also a risk towards others. Very quickly, you get a stamp like ‘this one has autism and everything which goes wrong will have to do with that.’ They will already look differently at our child. I would find it regrettable when a teacher immediately sees the document that mentions the autism diagnosis and therefore changes his practices without truly knowing the child.”*

This links to a second issue with disclosing their child’s autism diagnosis to others. Parents reported or feared that autism is too often interpreted in a stereotypical, negatively connotated and all-encompassing way. While the diagnosis might give indeed a new perspective on the child’s functioning, which may help understanding certain behavior, several parents critically positioned themselves towards such one-size-fits-all interpretations of autism as these can become overly dominant and overshadow their child’s unique characteristics and strengths.

*Mother (IV 7, siblings group): “His diagnosis [referring to older sibling] is known at school. But during parent-teacher moments and care coordination meetings, they often start talking about his weaknesses and difficulties, and only at the end some positive points are highlighted. (…) Father: While this is not even always necessary. As parents, it also really nice to hear that your child is simply doing well; rather than: ‘we do not notice so much that autism affects him that badly?’”*.

Based on his intuitions and on the experience with the autistic daughter of a close friend, one father from the feeding difficulties group (IV 6) phrased the discussion on the benefits and risks of labelling a child as follows:

*The biggest disadvantage (of obtaining an autism diagnosis) is the pigeonholing (…). As parents we could start looking for solutions within that category of autism. But outsiders, they never think broadly within categories. People always think they know what it’s like to be autistic, while it is such a broad spectrum. (…) But then, hey, in case our daughter would be autistic, whatever people think of that, it would not interest me, if we can turn that (diagnosis) into something positive.”*

Some parents did indeed share their intentions or experiences to break negative connotation and stereotypical interpretation of autism, for example by talking openly about autism to people around them. A father from the preterm group (IV 10), trained as a nurse, said that *“if you are overly protective of your child, then everyone will look at your son like: oh, he has autism and this and that. Some people in our environment will definitely panic. And then it is our job to say, act normally, these are the things that you need to take into account. So, I think that the stigmatization is something you have control over yourself (as parent).”*

Some other parents, mainly from the sibling group, also addressed this same topic of trying to resist stereotypical understandings of autism by talking openly about autism to their family. A mother of three (IV 2), of whom the oldest two already had an autism diagnosis said that “*autism is simply present (within our family conversations). Very normal, very ordinary. So, let’s not be silly, no taboos. And maybe that is the biggest advantage of having an early diagnosis.”*

### The “right” Words

Throughout the interviews, parents across groups and across levels of experience with autism were very regularly reconsidering the words they used as they wanted to refer to autism as a condition.

*Mother (IV 12, preterms group) “It is often not easy for parents, because there is something wrong. Although that is maybe not correct to say so, but it isn’t a normal child either (…). Actually, it is not okay to say that ‘something is wrong’, according to me. Because everyone is unique. But on the other hand, there is the standard, and then you have children or people who fall outside of that standard. So, that does not mean that something is wrong, but yeah…”*.

*Mother (IV 2, sibling group) “Our home guidance practitioner once said, some researchers work on the idea that autism is not a diagnosis but rather that there are two kinds of brains in the world. So, I would find it really cool that one day, it would turn out that there is nothing abnormal about our children, but that it is… Uhm… No disease… Father: Like you are either a boy, or a girl, you are either autistic or you’re not.”*

## Discussion

We initiated this interview study with questions on how parents would weigh potential benefits and risks of early autism detection programs. Would parents indeed think that earlier is always better? However, when we analyzed parents’ responses, it turned out that, rather than clear lists of benefits and risks, we had collected stories of parents drawing on both past and current experiences, and on expectations for the future. Overall, these data represented complex, nuanced parental positions towards early detection and diagnosis. This unexpected turn of the interview data complicated answering our initial research questions in the way we presumed. Nevertheless, the findings do offer valuable empirical insights for the ethical debate on early autism care.

First of all, the expectations and experiences shared by the parents in this study reaffirmed that being a parent to a child who differs from the developmental norm is often a challenging task in many respects. In accordance to the existing qualitative literature on prediagnostic experiences (e.g. Jacobs et al., 2020), parents discussed aspects of misunderstanding their child, feelings of guilt, frustration, lack of self-perceived parenting competency, and not being recognized as “good parents” by others. One way to summarize these challenges experienced or expected in a pre-diagnostic phase, is that they could not be the parents they wanted to be for their child. Against this backdrop, nearly all participating parents held a positive overall position towards diagnosing autism at a young age, as they expected or experienced this diagnosis would support them in their challenges.

Moreover, the value of such an autism diagnosis at a young age seemed most of all *relational* in nature. Following an autism diagnosis, parents described how it provoked a “*click*”, changing how they thought about and engaged with their child, how they perceived themselves as parents, and how they related to third parties such as extended family and other caregivers. Importantly, parents described that an official diagnosis could serve as a justification to think, feel and behave differently as a parent, compared to what they initially thought of as “normal” parenting. On the other hand, parents reported that deviating from the norm and explicitly mobilizing the diagnostic label generate new tensions as well, flowing from narrow or stereotypical views on autism held by relevant people in their child’s life.

### Navigating the Ab/Normal Binary

Overall, our findings underscore parents’ complicated position of navigating between either of two spheres that are available in their societal context: the realms of “the normal” and “the abnormal”. However, both sides of this binary divide seem to come with a fairly rigid set of norms and expectations, not only in respect to the child’s development and behavior, but also regarding the conception of what it means to be a good parent. When these parents no longer feel comfortable in their role, the diagnostic label offers a way out of the expectations of “normal development” and “normal parenting”. Yet, even when the diagnosis is welcomed in this sense, parents tend not to settle down in the sphere of the abnormal either.

Some parents shared indeed how they did or intended to work through the tensions generated when mobilizing the diagnostic label of autism. This involves a careful reflection on when and to whom to disclose their child’s diagnosis to obtain certain accommodations, and when not to speak about it to avoid negative or unhelpful reactions. In accordance with McLaughlin & Goodley (2008), we could describe such goal-oriented choosing between various discourses without being fixed to one or the other, as “strategic agency” on the part of parents. Moreover, when the time is right, some parents explicitly choose to talk openly and positively about autism at an early age within their household and with relevant others. This finding confirms Russell & Norwich’s (2012) earlier observations of parents taking a pro-active position in a post-diagnostic phase to destigmatize or normalize autism. Lastly, some parents explained how a “new normal” came about within their family as their adapted, more autism-friendly parenting practices and choice of family activities simply became part of their routines. Be it at micro-scale, we can interpret this as parents engaging in a sort of “politics of practice” (Hart, 2014), redefining the dominant norms on development and parenting that reign outside of the family by means of everyday practices. To some extent, this kind of politics of practice was also reflected by parents struggling to find the right words and correcting themselves in the terms they used to refer to autism. By referring to autism in terms of deficits and disease, they echoed the dominant discourse in society, but by trying to reformulate they also showed motivation to resist and change this discourse into a more neutrally phrased one.

We believe the latter observation sheds a new light on the position of parents in autism and autistic communities. In our study, we have seen that parents are simultaneously *subjected to* the challenges raised by a binary normal/abnormal ideology centered around neurotypicality as the norm, while they are also *subjects* themselves who take an active role in undermining this divide. This contrasts the oft-cited histories of pro-cure parental advocacy groups which have been often perceived by autistic self-advocates and the neurodiversity movement as their political adversaries (Pripas-Kapit, 2020; Silberman, 2015; Sinclair, 1993; Waltz, 2013). Rather, our findings suggest that parents as well do experience a position of “otherness” and the perception of not fitting into society’s expectations (Ryan & Runswick-Cole, 2008). So, next to autistic people themselves, parents of autistic children do seem to endure certain negative effects of a neurotypical-dominated society in their struggle to be a good parent. Based on this experiential overlap, we expect that ideas and discourse of the neurodiversity movement might be valuable for parents as well.

### Implications for the Ethical Debate on Early Autism Care

The goal of this study was to enrich the ethical debate on early autism detection and diagnosis with the perspectives of parents of a potentially autistic child. Based on our findings, there are at least three insights and implications for this debate.

First, the value of an autism diagnosis for parents seems to be context dependent. Rather than considering a diagnosis as an inherently good or bad thing, parents rather discussed how the diagnosis might be valuable within a given societal context and at a specific moment in their lives. Especially when parents experienced or anticipated they could not be “good parents” to their child, a diagnosis appears welcome to them. The timing at which parents reach this point does differ though. In line with our analysis, we suggest that a main determinant of this timing is whether parents and relevant others need a justification to accept the child’s developmental difference, and to engage in an adjusted pedagogical approach. As we have seen in the siblings group for example, while some parents wanted to have their child assessed as soon as possible, other parents indicated that the need to obtain an early diagnosis for their youngest child was lower compared to their older child with autism, as the norms within their family shifted over time on what counts as “normal” development and parenting.

This might imply that it could be more valuable to think in terms of a “timely” autism diagnosis, at least from parents’ perspectives, rather than thinking in terms of an early diagnosis at a fixed age as is often proposed in the context of universal or targeted screening programs. A timely diagnosis, rather than merely an early one, would do more justice to the experiences and expectations shared by parents in this study. Indeed, a diagnosis was not merely valued as the outcome of an abstract process of objectively determining individual autism characteristics of their child. Parents rather described the important relational functions of an autism diagnosis taking place in a specific context. In current clinical practice, providing such a timely diagnosis is not self-evident though. In our Flemish context for example, prioritization schemes help to speed up diagnostic assessments for autism under the age of two-and-a-half to three years, yet, waiting lists go up to two years for (pre)school-aged children and adolescents. This obviously undermines the idea of a timely diagnosis.

Also, these findings suggest that a “pre-symptomatic” detection of autism (from parents’ viewpoint) might not be welcomed by all parents. In such cases, prediagnostic experiences will differ markedly from the ones described in this study, potentially lessening the need for a diagnostic label to foster understanding, recognition, justification for altered parenting practices etc. As we discussed before, some parents wanted to know whether their child was autistic irrespective of experienced problems or needs. Other parents from the sibling group indicated that the function of a diagnosis was not the same anymore for a second or third child, as they already changed many of their parenting practices and expectations. Parents without much autism experience also indicated they wanted to be offered support at a time that they experienced issues, but than before.

Second, our analysis shows that parents of (potentially) autistic children are being negatively impacted by the conceptual ab/normal divide. On the one hand, a formal diagnosis seems necessary to justify a different parenting approach and to ask other caregivers to adapt their practices as well. On the other hand, mobilizing the diagnostic label often leads to stereotypical, narrow and negative interpretations of autism. Parents’ language use illustrated their ambivalent position, as they changed between and regularly corrected themselves, visibly struggling to use the “correct” terminology.

Moving away from this binary conceptualization towards a neurodiversity approach to autism might help tackle these experienced difficulties. Neurodiversity approaches understand autism as a one form of variation within a wide diversity of minds, functionings and ways of developing, be it a minority one, associated with strengths and vulnerabilities that are partly dependent on the accommodations society offers (Dwyer, 2022). When parents would be more familiar with neurodiversity approaches, we hypothesize that they would feel less pressured to stick to what they perceive to be the normal parenting practices. Accepting that there is a diversity of ways in which children develop could help parents to embrace as well that diverging parenting practices are needed and justified for their child, without necessarily needing an official diagnosis at that point. In post-diagnostic settings, parents might benefit from neurodiversity-discourse to discuss their child’s needs and accommodations in a more neutral way, rather than reinforcing a negative perception of autism as pathological condition by default, in need of treatment and remediation.

In a recent editorial in the journal *Autism*, Brown et al. launched a call to support a neurodiversity approach from the early start of clinical autism trajectories: “*it is critical that diagnosticians, who are often one of the first to frame autism for families, consider moving away from the medical model’s deficit-based story to a more balanced, neurodiversity-framed view of autism”* (Brown et al., 2021, p. 1171). Indeed, clinical practitioners seem well placed to acquaint parents with neurodiversity-thinking. This would obviously require adequate training for these practitioners, which could be extended as well to practitioners at well-baby visits, caregivers in child day care, and teachers. All these professionals play some role (formal or informal) in noticing (and communicating) a child differs from the developmental norm and/or are involved in implementing an autism-friendly environment once a diagnosis is established.

Lastly, our findings suggest that important aspects of why parents value an autism diagnosis for a young child are related to the actions they undertake as parents themselves. Of course, we have found that a diagnosis changes the personal state of affairs for parents, such as deflecting blame and providing a better understanding of their child. Yet, we have seen as well that parents mobilize the diagnosis to change the societal state of affairs as well, via what we have referred to as politics of practice. This way, an autism diagnosis does clearly not only function as a descriptive or a prescriptive term, which sets in stone how things are or should be; an autism diagnosis seems to be a *productive* label too, which opens space for parents to start doing things differently and work towards autism-friendly environments. Parents are, thus, not simply subjected to the diagnosis and the professional advice which follows, but clearly also subjects themselves playing an active role in putting the diagnosis to work and turning it into something of value in their lives.

This finding might inspire researchers and practitioners to reshape the kind of support offered to parents. Now, post-diagnostic services for parents are either rather descriptive, such as psycho-educational sessions, or largely prescriptive in nature, such as parent-mediated early intervention programs. Based on our findings, it seems valuable to reflect on how such services can also gain a “productive” edge and support parents to think critically about raising an autistic child within a neurotypical society.

### Strengths and Limitations

With this study we aimed to contribute empirically to the urgent debate on the ethics of early autism detection, diagnosis and intervention. In contrast to earlier qualitative studies embedded in prospective infant sibling studies, our inquiry differs in terms of methodology, positionality and goals (Achermann et al., 2020; MacDuffie et al., 2020). We opted for full-fledged in-depth interviews with both parents (when possible) conducted at their home, using open-ended questions rather than for a tightly structured interview administered during the study visit. Also, our aim was not to evaluate parents’ satisfaction of and suggestions for early detection research practices, but rather to engage with them in a critical reflection on early detection from their proper perspective. Lastly, the first and last author of this manuscript were only engaged in the ethical work package of the TIARA study, and not in other parts of data collection and analysis. This way, there was more space to reflect on the goals and methods of such early detection research, compared to earlier work. Despite being time-intensive, the QUAGOL methodology for data analysis proved to be apt to handle the data generated with this diverse group of parents. Due to its case-oriented approach, constant comparison within and between cases, and its data-generated codes, we managed to tap well into common threads of the fourteen interviews, while also managing to make comparisons between the subgroups (Dierckx de Casterlé et al., 2021).

Finally, we want to point out this study’s limitations regarding the specificity and generalizability of the findings. Some of our findings, such as those described in Theme 1 are not entirely specific for *early* autism detection and diagnosis and confirm findings of previous qualitative work reporting on the experiences of parents of school-aged children and adolescents on the autism spectrum (Jacobs et al., 2020; van Esch et al., 2018). As we mentioned before, we could interview a very interestingly situated group of people as they represent potential early adopters of targeted, early autism detection, be it in a research setting. Obviously, this group does not represent all possible parents who might be approached in a future universal autism screening program: all interviewees were white and relatively highly educated. Also, many of our participants had some relevant experience with autism, while some did not at all. By consequence, our findings do not only reflect lived experiences of parenting a child *at increased likelihood* for autism, as we set out in the initial research questions. Instead, our findings also reflect some experiences in parenting an (older) autistic sibling, and parents’ expectations about what might happen in the future. Moreover, our interviewees might have had a positive baseline attitude towards detecting autism early in life, as they previously consented to take part in TIARA and agreed to be interviewed for this study as well. At the same, we have learned that this group of parents held nuanced and even critical opinions regarding the value of early autism detection as described in Theme 2 and 3.

## Electronic supplementary material

Below is the link to the electronic supplementary material.

Supplementary Material 1

## Footnotes

1 Despite ongoing debates, also within autistic communities worldwide, we opted to use autism-first language in this manuscript in line with preferences of a majority of English-speaking autistic people in Western countries (Keating et al., 2022). We have retained person-first language in quotes as they occurred in the interviews. This way we want to do justice to our participants’ own words (in Dutch and also to illustrate diverging opinions in Dutch-speaking regions, where person-first language is preferred by most people on the autism spectrum (Buijsman et al., 2022). We opted to use “autism” instead of “autism spectrum disorder” or “ASD” since the latter two options imply an inherent coupling of autistic features to distress or pathology. To the contrary, the term “autism” provides more space to capture the wide set of autistic, lived experiences that participants shared with us, whereas the clinical term “ASD” is more strictly delineated by its diagnostic criteria.

2 In our wider research project on the ethics of early detection and intervention, we are currently conducting a separate qualitative study on the topic with autistic adolescents.

3 The phrasing ‘at-risk for autism’ is widely used in research settings. We support the move away from such terminology as this frames autism as a threat or as a condition to be prevented. Although this phrasing is part of the acronym, the consortium now prefers more neutral language such as ‘increased likelihood’ for autism (Bottema-Beutel et al., 2021; Fletcher-Watson et al., 2017).

4 Increased likelihood for autism is here thus understood as a heightened empirical chance to develop autism based on well-studied determinants of family history and gestational duration (McDonald & Jeste, 2021). Establishing this likelihood in infants, and inclusion in the cohort study, was thus not based on potential presentation of early autism characteristics. Infants with feeding difficulties were a less-well studied group at the start of the cohort study, this group was included in the design to verify an earlier hypothesis on the relation between early, persistent and medically insufficiently explained feeding difficulties as a potential prodromal and/or co-occurring of autism (Field et al., 2003).

5 **Table 1:** Participants’ demographics

**Interview number**	**Group**	**Gender**	**Highest educational attainment**	**Ethnicity**	**Autism experience**	
**1**	sibling	woman, man	sec., postsec.	white (x2)	parents	
**2**	sibling	woman, man	postsec., sec.	white (x2)	parents	
**3**	feeding difficulties	woman, man	postsec., sec.	white (x2)	no explicit experience	
**4**	sibling	woman, man	postsec., sec.	white (x2)	parents	
**5**	feeding difficulties	woman*	postsec.	white	teacher in regular secondary education	
**6**	feeding difficulties	woman, man	postsec., postsec.	white (x2)	befriended couple has autistic daughter	
**7**	sibling	woman, man	postsec., postsec.	white (x2)	parents	
**8**	sibling	woman, man	postsec., postsec.	white (x2)	parents	
**9**	sibling	woman*	postsec.	white	parent, mother’s sister is autistic	
**10**	preterm	woman, man	postsec., postsec	white (x2)	father is trained as a nurse	
**11**	feeding difficulties	woman, man	postsec., sec.	white (x2)	mother’s sister is autistic	
**12**	preterm	woman, man	sec., sec.	white (x2)	no explicit autism experience	
**13**	preterm	woman, man	postsec., postsec.	white (x2)	mother has professional experience in autism home guidance service, father is teacher in regular secondary education	
**14**	sibling*	woman, man	postsec., postsec.	white (x2)	parents	

**sec**.= secondary (high) school diploma; **postsec**.= postsecondary certificate, diploma or degree

***** One parent couple from the sibling group (IV 14) decided to cancel their participation in the TIARA study due to the long commute to the research center. Yet, they still agreed to take part in the interview study. One couple recently split up, and for another one the father was ill on the day of the interview. Two interviews were, thus, conducted with mothers only (IV 5 and 9). The TIARA study involves two baby labs, one in Ghent and one in Leuven. For this interview we recruited only among participants attending the lab in [city 2] for reasons of feasibility. In total, 87 participants have been assessed here. During the recruitment window for the current interview study, twenty parent couples have been asked to participate in the interview as they happened to have an appointment at the baby lab at that time.
