# Supplementary material for: The Earlier, the Better? An In-Depth Interview Study on the Ethics of Early Detection with Parents of Children at an Elevated Likelihood for Autism
Source: J Autism Dev Disord. 2023 Sep 26;54(11):4130–44. doi: 10.1007/s10803-023-06139-8 (PMC11461763; doi:10.1007/s10803-023-06139-8)
Supplement: Supplementary file 1 — Supplementary Material 1 [file 10803_2023_6139_MOESM1_ESM.docx]

## Supplementary material: interview guide

- Why did you enroll for the TIARA study?
- How do you experience being a research participant in this study?
- What are your expectations for the outcome of the study?
- What did you experience when you first realized that your child was in an ‘at risk’ group for autism?
- What does ‘having an autism diagnosis’ mean to you?
- What kinds of possible benefits do you think an early diagnosis of autism would have?
- What kinds of possible risks do you think are associated with an early diagnosis of autism?
- Do you think you will tell your child, when he or she can understand the issues at hand, that she was part of this study

N.B. *The second and third topic served internal purposes to evaluate whether TIARA study participation in itself could be improved. The last question was asked in the initial interviews but generated little responses in relation to our research questions. For these reasons, answers to these responses are no part of this manuscript.*
